# Retraction Note: Predictors of depression among school adolescents in Northwest, Ethiopia, 2022: institutional based cross-sectional

**DOI:** 10.1186/s12888-024-05645-y

**Published:** 2024-03-12

**Authors:** Aklile Tsega Chekol, Mastewal Aschale Wale, Agmas Wassie Abate, Eyerusalem Abebe Beo, Eman Ali Said, Berhan Tsegaye Negash

**Affiliations:** 1 Department of Nursing, College of Medicine and Health Sciences, Hawassa University, Hawassa, Ethiopia; 2 Department of Psychiatry, Dr. Ambachew Memorial Hospital, Amhara Regional Health Bureau, Eyerusalem Abebe Beo, South Gondar, Ethiopia; 3 Department of Midwifery, College of Medicine and Health Sciences, Hawassa University, Hawassa, Ethiopia

**BMC Psychiatry (2023) 23:429**

10.1186/s12888-023-04899-2.

The Editors have retracted this article after concerns were raised. After an investigation, the publisher found that the data for this study was also presented in the thesis [1] by the corresponding author. Further investigation by the Publisher found that the study in the thesis included a total of 578 participants from 4 schools (Fig. 2) and the study in this article included a total of 584 participants from 6 schools (Fig. 2), suggesting some of the participants were different in both studies. However, the Publisher noted that data in Tables 1 and 3 of the thesis [1] appear identical to data presented in Tables 1 and 4 of this article, despite having data from some different participants. The authors were not able to explain these differences satisfactorily.

The Editors therefore no longer have confidence in the reliability of the data presented in this article.

None of the authors have responded to any correspondence from the editor/publisher about this retraction.
